# Oxidative Stress From Exposure to the Underground Space Environment

**DOI:** 10.3389/fpubh.2020.579634

**Published:** 2020-10-21

**Authors:** Hongbiao Yu, Yijie Gao, Rong Zhou

**Affiliations:** ^1^Key Laboratory of Birth Defects and Related Diseases of Women and Children (Sichuan University) of Ministry of Education, Department of Obstetrics and Gynecology, West China Second University Hospital, Sichuan University, Chengdu, China; ^2^Department of Anesthesiology, Nanchong Central Hospital, Nanchong, China

**Keywords:** oxidative stress, underground space, hypoxic, low background radiation, environment, toxic particles, organism

## Abstract

There are a growing number of people entering underground spaces. However, underground spaces have unique environmental characteristics, and little is known about their effects on human health. It is crucial to elucidate the effects of the underground space environment on the health of humans and other organisms. This paper reviews the effects of hypoxia, toxic atmospheric particles, and low background radiation in the underground space environment on living organisms from the perspective of oxidative stress. Most studies have revealed that living organisms maintained in underground space environments exhibit obvious oxidative stress, which manifests as changes in oxidants, antioxidant enzyme activity, genetic damage, and even disease status. However, there are few relevant studies, and the pathophysiological mechanisms have not been fully elucidated. There remains an urgent need to focus on the biological effects of other underground environmental factors on humans and other organisms as well as the underlying mechanisms. In addition, based on biological research, exploring means to protect humans and living organisms in underground environments is also essential.

## Introduction

Available surface space is decreasing, and ground resources are being exhausted (1), which causes unprecedented challenges for the sustainable development of human beings. People must go underground to explore space and exploit resources. Thus, human history has progressed to an era of the development and use of underground spaces (2). Underground space refers to a space below the surface of the earth or belowground (3) that is utilized in various forms, such as human construction, including parking lots, subways, tunnels, commercial buildings, and laboratories, mines, including coal, gold, and uranium mines, and even underground animal burrows/caves. Underground space has unique environmental characteristics due to its particular geographical location; a typical characteristic is the enclosed environment below the surface of earth (4), which is prone to cause decreased oxygen concentrations and increased carbon dioxide concentrations (5, 6), elevated humidity and temperature (7), increased toxic particle concentrations (8), and accumulation of radioactive radon (9). The underground space environment is also characterized by a low dose of cosmic radiation and lack of sunshine due to shielding by thick soil and rock (2). In addition, the characteristics of different underground space environments vary due to their different depths, rock compositions and application purposes.

**Figure 1:**
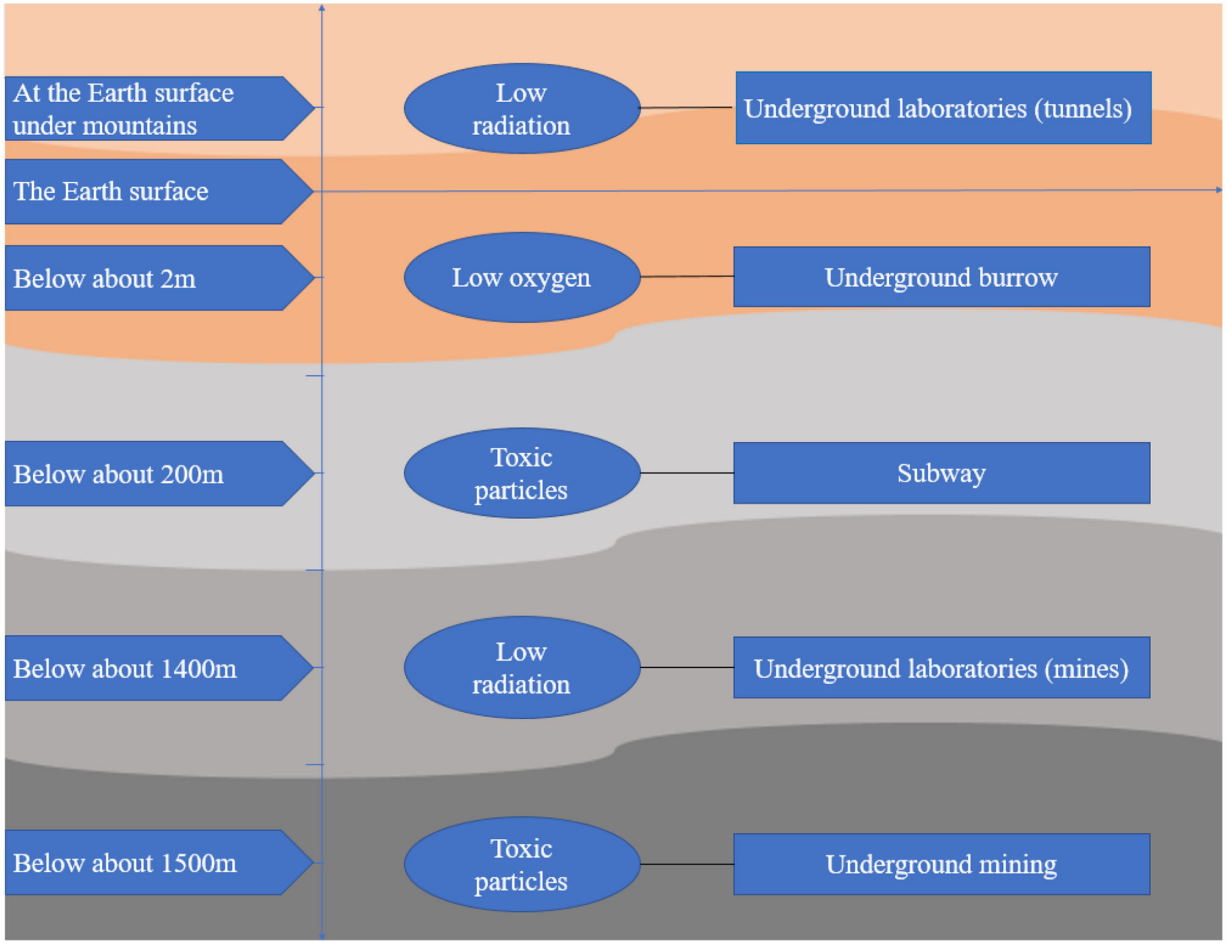
Sources of oxidative stress from exposure to the underground space environment.

All the features in the underground space environment pose challenges to the process of humans going underground. However, few studies have focused on the effect of the underground space environment on the health of humans and other living organisms. This review searched articles about the effect of environmental factors in underground space on organisms and summarized these articles from the perspective of oxidative stress in order to draw more attention to individuals staying in the underground space and help outline the emerging understanding of this area for those interested in contributing to future research. The searched resources about oxidative stress due to exposure to the underground space environment are shown in Figure 1.

## Oxidative Stress

**Figure 2:**
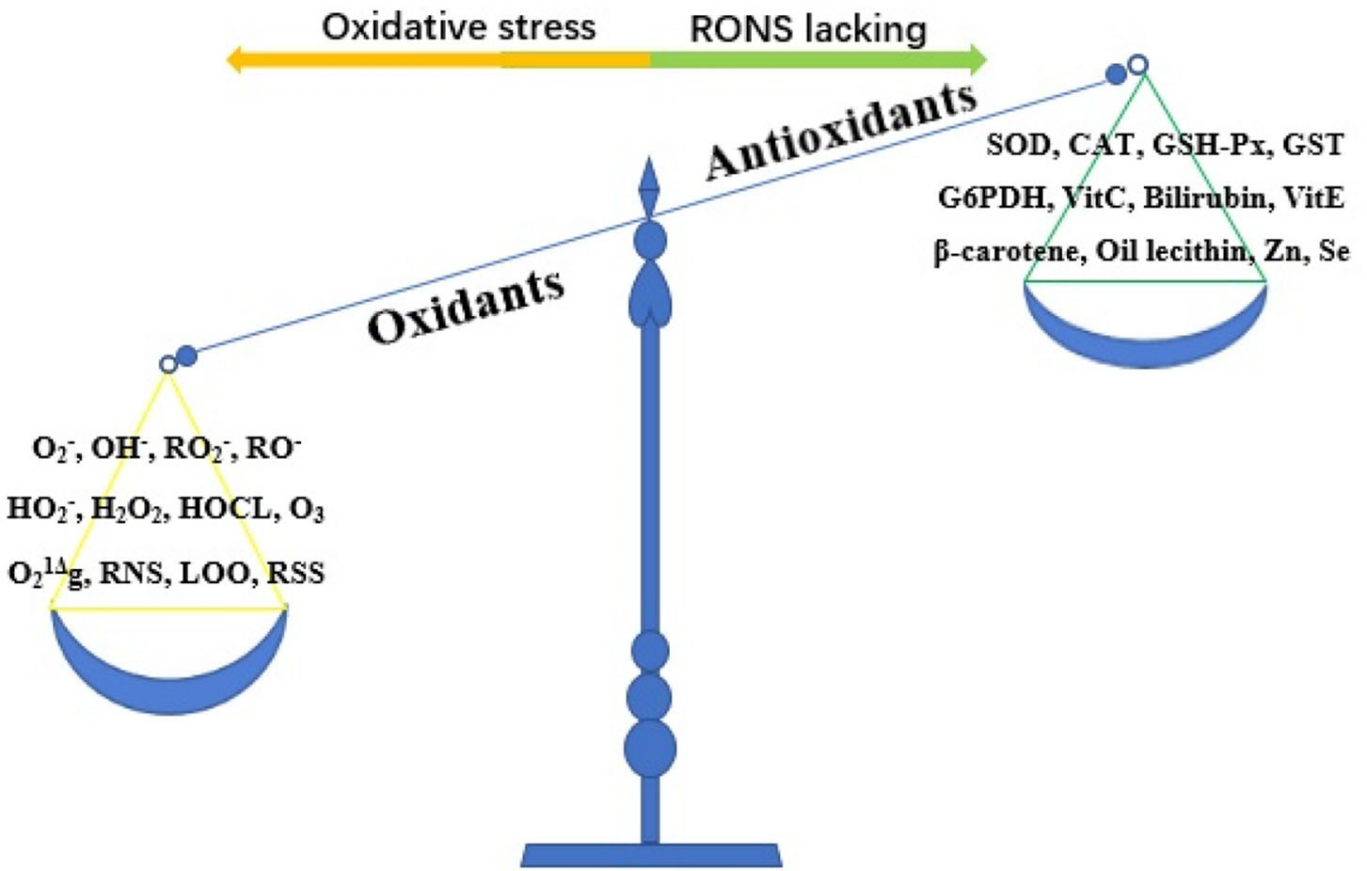
The imbalance of oxidant and antioxidant causes oxidative stress. RONS, reactive oxygen and nitrogen species; ${\text{O}}_{2}^{-}$, superoxide; OH^−^, hydroxyl; ${\text{RO}}_{2}^{-}$, peroxyl; RO^−^, alkoxyl; ${\text{HO}}_{2}^{-}$, hydroperoxyl; H_2_O_2_, hydrogen peroxide; HOCL, hypochlorous acid; O_3_, ozone; ${\text{O}}_{2}^{1\text{Δ}}$g, singlet molecular oxygen; RNS, reactive nitrogen species; LOO, lipid peroxide free radical; RSS, reactive sulfur species; SOD, superoxide dismutase; CAT, catalase; GSH-Px, glutathione peroxidase; GST, glutathione-S-transferase; G6PDH, glucose-6-phosphate dehydrogenase; Vit C, vitamin C; Vit E, vitamin E; Zn, zinc; Se, selenium.

Oxidative stress refers to the imbalance between oxidants and antioxidants caused by a variety of endogenous or exogenous factors, which favors oxidants (10) (Figure 2). Oxidative stress is essential for life and responsible for the death of living organisms. Oxidants, also known as reactive oxygen species (ROS), are generated as metabolic byproducts by biological systems, and ROS include unstable radicals, such as superoxide (${\text{O}}_{2}^{-}$), hydroxyl (OH^−^), peroxyl (${\text{RO}}_{2}^{-}$), alkoxyl (RO^−^), and hydroperoxyl (${\text{HO}}_{2}^{-}$), as well as non-radicals such as hydrogen peroxide (H_2_O_2_), hypochlorous acid (HOCL), ozone (O_3_), and singlet molecular oxygen (${\text{O}}_{2}^{1\text{Δ}}$g) (10, 11). In addition, oxidants also contain reactive nitrogen species (RNS), lipid peroxide free radical (LOO), and reactive sulfur species (12). Of the oxidants, ROS are the most widely studied type and play a key role in regulating cell function and biological processes, including the activation of several transcriptional factors, protein phosphorylation, apoptosis, immunity, and differentiation (13). The burden of reactive oxygen and nitrogen species (RONS) production is largely counteracted by an intricate antioxidant defense system that includes the enzymatic scavengers superoxide dismutase (SOD), catalase (CAT), and glutathione peroxidase (GSH-Px) (14). Other antioxidant enzymes include glutathione-S-transferase (GST) and glucose-6-phosphate dehydrogenase (G6PDH) (10). Antioxidants also contain non-enzymatic antioxidants, which refer to the protein compounds that interact with RONS and terminate the free radical chain reactions, including ascorbic acid (vitamin C), bilirubin, α-tocopherol (vitamin E), β-carotene, oil lecithin, selenium, and zinc (15, 16). When maintained at low or moderate concentrations, free radicals have several beneficial effects on living organisms (13). When the concentration of free radicals exceeds a certain limit, the condition is extremely unstable because these free radicals contain unpaired electrons and easily react with neighboring molecules, inducing new free radical generation; this process can stimulate free radical chain reactions, subsequently leading to oxidative damage of DNA, proteins, lipids, and carbohydrates and producing various pathophysiological effects by changing the function of these macromolecules (13, 17). To date, oxidative stress has been proven to be involved in the pathological processes of numerous human diseases, such as Parkinson's disease, Alzheimer's disease, atherosclerosis, cancer, major depression, diabetic nephropathy, pregnancy-related diseases, renal disease, and cardiovascular disease (12, 18).

Environmental factors from various artificial and natural sources, including atmospheric pollution (quartz, coal mine dust, and asbestos), heavy or transition metals (copper, mercury, lead, iron), hypoxic conditions, certain drugs, chemical solvents, cigarette smoke, and radiation, may serve as exogenous sources of oxidants and cause metabolic changes in organisms that either increase the production of RONS or decrease the production of antioxidants, causing oxidative stress (13, 19–21).

## Underground Space Environmental Factors and Oxidative Stress

### Underground Hypoxic Environment and Oxidative Stress

In the absence of effective ventilation, the exchange of air between closed underground spaces and the surface of earth is reduced, which eventually yields a state of hypoxia in the underground space environment (5, 6). Acute or chronic exposure to environments with reduced availability of oxygen is known to elicit oxidative stress in living organisms (22, 23). Although no report has investigated oxidative stress caused by the hypoxia in underground environments in humans, we have found several reports of oxidative stress in animals caused by hypoxic factors in underground burrows.

Underground burrows can reach an approximate depth of 2 m below the surface and are hypoxic-hypercapnic environments (5, 24). The oxygen (O_2_) concentration in burrows fluctuates with changes in soil permeability, temperature, tunnel depth and diameter, rainfall, and soil texture, further reducing the O_2_ concentration (25–27). In northern Israel, the minimal O_2_ levels in the burrow of *S. carmeli*, a kind of rodent that lives underground for its entire life, were as low as 7.2% (lower than the O_2_ concentration of 21% at the surface) (6). This low-oxygen environment may cause or upregulate oxidative stress in cave-dwelling creatures. Investigation of the garter snake found that the cave-hypoxia environment increased SOD activity in skeletal muscle and liver (increased by 59–118%, respectively) and gave rise to a 57% increment in GSH levels in the muscle (28). In rodents, the Harderian gland (a lacrimal gland) of *Spalax ehrenbergi*, which lives in underground caves, exhibited higher activity of antioxidant enzymes, including SOD, CAT, and glutathione reductase (GR), than *Syrian hamster*, which lives on the ground, and the difference in SOD activity was the most obvious; this can be understood as an adaptive antioxidant strategy to avoid deleterious effects caused by a hypoxic environment (29). Furthermore, another study found that there were higher transcript levels of antioxidant genes (*Cat, Gpx, Gst, Hmox1, Sod1*, and *Sod2*) and the transcription factor nuclear factor (erythroid-derived 2)-like 2 (*Nrf2*) in *Spalax* tissue (living underground) than in rat tissue (living ground) (30). All these studies revealed that the hypoxic environment in underground caves causes oxidative stress in cave-dwelling animals from the perspective of increased antioxidant activity and revealed a mechanism of adaption of cave-dwelling animals to the hypoxic environment. This is the adaptation of the antioxidant defense system that allows cave-dwelling animals to survive for days, weeks, or months in hypoxic underground environments. It must be noted, however, that not all living organisms can survive in low-oxygen environments for a long time, especially human beings, and a few minutes of hypoxia is lethal; thus, it is essential to maintain sufficient oxygen supply in the underground space environment to sustain life.

### Underground Atmospheric Environment and Oxidative Stress

#### Subway Systems and Oxidative Stress

Since the first subway in the world was built in London in 1863 (31), subway systems, as a typical type of urban underground space, have been expanding in depth and scope. Currently, subway systems have reached depths of ~200 m underground (Pyongyang metro station, North Korea) and a total number of more than 150 worldwide (32, 33). In 2012, more than 160 million passengers spent considerable time in subway systems around the world (33); by 2017, nearly 53.8 billion people traveled through subway systems (34), and the total number is still growing. Subway systems, therefore, are characterized by dense population, frequent train and population movement, and relatively closed space, which makes the concentration of atmospheric particles in the subway significantly increase (8, 35–37). Studies have shown that the concentrations of particular matter (PM) 10 and PM2.5 in subway systems were ~20–50% higher than those in the aboveground environment (38, 39) and were affected by train running, crowd activity, season, and outdoor climate (38, 40–42). Atmospheric particles in subway systems contain high concentrations of metal compounds with the potential for oxidation, including manganese (Mn), chromium (Cr), titanium (Ti), iron (Fe), copper (Cu), zinc (Zn), nickel (Ni), and molybdenum (Mo), as well as organic compounds, such as polycyclic aromatic hydrocarbons (PAHs), which come from subway tracks, wheels, chain rings, brake pads, and pantographs (33, 35, 37, 43, 44). The elevated particles increased commuter exposure to them; for example, subway personal PM exposure increased by 3%, Fe exposure increased by nearly 200%, Mn exposure increased by 60%, and Cu exposure increased by 40% (45, 46). When people are exposed to the subway environment, these particles enter the alveoli with breathing and even enter the circulatory system through respiratory membranes, causing harm to human health. Previous reviews have focused on the health effects of subway particles but have not described oxidative damage in detail (47).

The oxidation potential of atmospheric particles in the subway leads to oxidative stress by increasing intracellular ROS (48). These studies are summarized in Supplementary Table 1. A survey of 81 subway workers in Tehran showed that high concentrations of Fe, Mn, and Cr in subway atmospheric particles may lead to excessive oxygen free radical generation in workers exposed to a subway environment, resulting in a significant increase in urinary 8-hydroxy-deoxyguanosine (8-OHdG) (49). In contrast, another study found no significant differences in oxidation biomarkers among subway workers, bus drivers, and suburban office workers, even though the concentrations of Fe, Mn, and Cr in the subway air were more than 100 times higher than those in aboveground air (50). A comet assay was used to detect oxidative DNA damage in the human lung cell line A549 induced by particles extracted from the Stockholm subway. The results showed that subway particles caused obvious oxidative DNA damage, as manifested by a significant increase in DNA breaks and an increased level of 8-oxo-2′-deoxyguanosine (8-oxodG) (51). Data from Karlsson et al. (52) showed that particles from the Stockholm subway were more toxic than urban street PM in causing DNA strand breakage (approximately eight times) and inducing oxidative stress (four times) in A549 cells, indicating the involvement of high iron levels in subway particles, mainly in the form of magnetite (Fe_3_O_4_). Then, the team further compared the toxicity of subway particles and magnetite particles (mainly Fe_3_O_4_) to A549 cells and found that the genotoxicity of subway particles could not be explained by the main component (magnetite), water-soluble metals, or intracellular mobilized iron, which was most likely caused by oxidative stress induced by highly reactive surfaces (53). However, Spagnolo et al. (37) found significantly increased concentrations of iron and other transition metals in PM from a subway platform, and intracellular ROS in non-small cell lung cancer cell lines (NCI-H727 cells) were correlated with the concentrations of some transition metals in PM, such as Mn, Cr, Ti, Fe, Cu, Zn, Ni, and Mo. Moreover, these authors also found that the intensity of oxidative stress progressively declined as particle diameter diminished. However, another study held the opposite viewpoint, that is, fine particles had a more potent ROS production capacity than coarse particles, and 90–98% intracellular ROS were caused by fine particles with smaller diameters. It was also discovered that 94% of intracellular ROS in alveolar macrophages were correlated with water-soluble Fe (*R*^2^ = 0.77), Ni (*R*^2^ = 0.95), and organic carbon (*R*^2^ = 0.92) in subway particles (54). Furthermore, a study revealed that subway particles, regardless of size, contain similar concentrations of transition metals (Fe, Cu, Cr, Mn, and Zn) and produce ROS in a concentration- and size-dependent manner. Ultrafine particles (PM0.1), which may be derived from high-temperature processes, have a stronger ROS-inducing effect on primary bronchial epithelial cells (PBECs) due to their larger surface area/volume ratio and high metal content (55). Subsequently, this team further proved that fine and ultrafine particles generated more ROS than coarse particles and found that ultrafine particles produced ROS in PBECs by an iron-dependent mechanism and increased the expression of the intracellular antioxidant gene heme oxygenase-1 (HO-1) (56). In addition to the ion-dependent mechanism, Jung et al. (57) found that the organic extract (OE) induced a significantly elevated level of ROS and antioxidant (SOD and CAT) production in human normal bronchial cells (BEAS-2B) by exposing cells to the OE of subway PM10 for 24 h. In addition, these authors also found that OE caused a significant increase in DNA and chromosomal damage in CHO-K1 and BEAS-2B cells, suggesting that oxidative stress could be one of the major mechanisms responsible for genotoxic effects. The murine macrophage-like cell line RAW 264.7 showed significant lipid peroxidation and intracellular ROS formation after 18 h of exposure to 100 μg/ml particles from the Stockholm subway, and there was a concentration–effect relationship between the ability of subway particles to generate ROS, but the components of the particles were not provided (58). Some researchers compared the biological effects of PM10 from two sites of the same subway system and found that although the components of PM10 from two sites were different, both induced significant oxidative stress (HO-1 expression) in RAW 264.7 cells after exposure for 24 h, while there was no significant difference in HO-1 between the cells treated with particles from the two sources. Furthermore, the authors observed significantly increased oxidative stress in the lungs of C57BL/6 mice administered PM10 for 8 h (59). Gali et al. (44) observed that intracellular ROS were positively correlated not only with transition metals in subway particles but also with organic and elemental carbon (OCEC) in the particles (*r* > 0.85). Moreover, coarse particles can induce intracellular ROS production more than fine particles.

The factors influencing the concentration of subway particles include station depth, construction date, ventilation rate, proportion of friction of regenerative braking, train frequency, wheel type (rubber vs. steel), and the presence or absence of platform edge doors and/or air conditioning in subway cars and stations (50). Thus, the atmospheric quality, composition, and oxidation potential of particles in different subways are varied, and the biological oxidative stress from subway particles may not be consistent. The results obtained may not be applicable to all subway systems. It is necessary to regularly monitor and assess the impact of subway particles on organisms to ensure the safety of subway systems.

#### Underground Mining and Oxidative Stress

Increasing numbers of people are participating in underground mining, and underground mining is being driven to a deeper level in response to the mineral supply crisis, especially underground coal mining. Currently, underground coal mining has reached a depth of ~1,500 m (2). Occupational exposure to closed underground coal mines leads to workers being subjected to long-term exposure to high concentrations of coal dust particles (containing metal compounds, inorganic compounds, and polycyclic aromatic hydrocarbons) (60, 61). These factors pose a major threat to the occupational safety and health of workers, causing bronchitis, pulmonary fibrosis, pneumoconiosis, lung cancer, and even other diseases (62). At present, studies have confirmed that oxidative stress is the main pathogenesis of workers' diseases caused by occupational exposure to underground coal mines (63, 64).

Some researchers have focused on the oxidative stress induced by underground coal mines. The studies are summarized in Supplementary Table 2. An investigation of the relationship between coal dust exposure and oxidative stress found that subjects directly or indirectly exposed to coal dust exhibit oxidative stress. Moreover, underground coal workers showed significant differences in GSH-Px activity compared with healthy subjects and other coal dust-exposed subjects (65). Perrin-Nadif (66) first reported that erythrocyte Cu^++^/Zn^++^ SOD activity was significantly higher in underground coal workers than in surface coal workers. The team then investigated 240 underground coal miners and confirmed that the production of ROS may be an important event in coal mine dust exposure and the severity of coal workers' pneumoconiosis (CWP). Moreover, these authors found that erythrocyte CAT and Cu^++^/Zn^++^ SOD activities were more closely related to recent exposure to high concentrations of dust than to cumulative exposure (67). Altin et al. (60) investigated the SOD, GSH-Px, and malondialdehyde (MDA) concentrations in underground workers at Zonguldak coal mines and discovered that oxidative stress caused by the production of free radicals and active oxygen metabolites existed in the early and late stages of CWP diagnosed by high-resolution computed tomography. Similarly, the investigation performed by Engelen et al. (68) found that erythrocyte GSH-Px levels were significantly reduced in underground coal workers with radiograph classifications of 0/1–2/1 CWP compared to underground coal workers without CWP. Moreover, the test results of oxidative stress indicators (CAT, GSH-Px, SOD, and lipid peroxidation) in bronchoalveolar lavage fluid of underground coal workers confirmed that the development of CWP and its progression may be correlated with oxidative stress (69). However, another study failed to confirm the correlation between CWP and oxidative stress by detecting the 8-oxodG/dG ratio of peripheral blood lymphocytes of underground coal workers and only found that the 8-oxodG/dG ratio in underground coal workers was higher than that in healthy subjects without coal dust exposure (70). By investigating the relationship between pneumoconiosis and gene polymorphisms of oxidative stress indicators (*MnSOD, GSTM1, GSTT1*, and *OGG1*) in retired underground coal workers, Zhai et al. (71) found that cumulative coal dust exposure, rather than genetic polymorphisms, was significantly associated with CWP. Yucesoy et al. (72) also found no statistically significant association between genetic polymorphism (*GST* and *MnSOD*) and pulmonary toxicity caused by underground coal dust exposure in individuals. However, a retrospective investigation by Nadif et al. (73) suggested that both genetic polymorphisms (*TNF* and *LTA*) regulating oxidative stress and underground coal dust exposure play a role in the development and precession of CWP in underground coal workers. A study validated the role of oxidative stress in pulmonary toxicity (pulmonary fibrosis and pneumoconiosis) induced by underground coal dust exposure in a rat model and confirmed the attenuating effect of the antioxidant erdosteine on this toxicity (63). Some researchers treated subjects exposed to underground coal dust with antioxidants (vitamin E 800 mg/day and vitamin C 500 mg/day) and found that oxidative stress biomarkers (contents of lipoperoxidation, protein carbonyls, α-tocopherol, GSH, GSH-Px, SOD, CAT, and GST) were restored to pre-exposure values after antioxidant supplementation (64). Contrary to the above findings, a study on surface coal workers, underground coal workers without pneumoconiosis, and underground coal workers with simple pneumoconiosis showed no significant difference in antioxidant enzyme activities (SOD, CAT, and GSH-Px) between surface workers and underground workers without pneumoconiosis, no significant difference in Cu^2+^/Zn^2+^ SOD activities between underground workers with simple pneumoconiosis and underground workers without pneumoconiosis (74).

In addition to the underground coal mining environment, other underground mineral mining environments also induced pulmonary oxidative damage. Nardi et al. (75) found that MDA levels were significantly increased in workers exposed to crystalline silica (working in underground mines) compared to non-exposed workers.

### Underground Low Background Radiation Environment and Oxidative Stress

All living organisms on earth are exposed to a relatively constant dose of ionizing radiation called natural background radiation (76–78). Due to the shielding effect of overburden, the underground space environment, especially the deep underground, exists in a state of low background radiation (79). In the past few decades, several deep underground laboratories have focused on the influence of low background radiation on living organisms (76, 78, 80–88). Among these, most have observed oxidative stress in living organisms exposed to low background radiation. These studies are summarized in Table 1.

**Table 1:** Underground low-background radiation environment and oxidative stress.

**Author/year**	**Location**	**Exposure subjects**	**Oxidative indicators**	**Study results**
Satta et al. (87)	LNGS, Italy	Chinese hamster V79 cells	GST, GSH-Px, GR, CAT, SOD	The activity of CAT, GSH-Px, and GR in cells cultured in low-background radiation environment were significantly increased, while the SOD activity decreased.
Carbone et al. (81)	LNGS, Italy	Human lymphoblastoid TK6 cells	SOD, CAT, Se-GSH-Px, CAT/SOD, Se-GSH-Px/SOD	The activity of CAT, Se-GSH-Px, and the ratio of CAT/SOD and Se-GSH-Px/SOD in the cells cultured under low-background radiation decreased significantly. After X-ray exposure, the Se-GSH-Px activity of cells cultured in low-background radiation environment decreased significantly.
Carbone et al. (80)	LNGS, Italy	Human lymphoblastoid TK6 cells	Se-GSH-Px/SOD ratio	After 1 Gy ion radiation exposure, compared with before culture, ROS scavenging ability of cells cultured in normal background radiation increased, while ROS scavenging ability of cells cultured in low-background radiation significantly disappeared (Se-GSH-Px/SOD ratio decreased).
Smith et al. (88)	WIPP, USA	Primary human lung fibroblast and bronchial epithelial cells	Hsp90, Hsp70, Hsp27	Hsp90 and Hsp70 were upregulated in low-background radiation environment.
Castillo et al. (82)	WIPP, USA	*S. oneidensis* and *D. radiodurans*	*katB, recA, oxyR, lexA, dnaK, SOA0154*	Upregulation of *katB, recA, SOA0154* genes in *S. oneidensis*, and the upregulation of *dnaK* in *D. radiodurans*.
Fratini et al. (83)	LNGS, Italy	Chinese hamster V79 lung fibroblasts	SOD, CAT, GSH-Px	The GSH-Px activity in the cells cultured between normal- and low-background radiation had a 4-fold difference, as well as significant differences in gene levels (*Gpx2, Gpx4*).
Castillo et al. (76)	WIPP, USA	*D. radiodurans*, and *S. oneidensis*	*KatB, oxyR, recA, lexA, dnaK, SOA0154, dps, gapdH*	Genes involved in oxidative stress *(katB, oxyR)* were upregulated in *S. oneidensis*, while *D. radiodurans* downregulated genes involved in oxidative stress (*dps* and *gapdH*).

*SOD, superoxide dismutase. CAT, catalase. GSH-Px, glutathione peroxidase. 8-OHdG, 8-hydroxy-deoxyguanosine. 8-oxodG, 8-oxo-2′-deoxyguanosine. GST, glutathione-S-transferase. GR, glutathione reductase. HSP, heat shock protein*.

Human lymphoblastoid TK6 cells grown over 6 months under low background radiation showed significantly decreased levels of selenium-dependent glutathione peroxidase (Se-GSH-Px), CAT enzymes, and the CAT/SOD and Se-GSH-Px/SOD ratios compared to cells grown under a standard background radiation, while SOD activity remained stable. The same research also found that the Se-GSH-Px activity of cells grown in low background radiation decreased significantly compared with that of cells grown in normal background radiation after acute exposure to 1 Gy X-ray radiation (81). The same researchers used the Se-GSH-Px/SOD ratio to detect the ROS scavenging capacity of human lymphoblastoid TK6 cells after acute exposure to 1 Gy ionizing radiation and found that the ROS scavenging ability of cells cultured in normal background radiation increased, while that of cells cultured in low background radiation disappeared (decreased Se-GSH-Px/SOD ratio), compared with that before culture (80). Smith et al. (88) observed the upregulation of the oxidative stress-related protein HSP70 in primary human lung fibroblast cells and bronchial epithelial cells as a result of growth in a low radiation background. By culturing V79 Chinese hamster cells in the Gran Sasso underground laboratory (LNGS, shielded by at least 1,400 m of rock overburden), Satta et al. (87) showed that after 9 months of culturing independent cell lines under standard and low background radiation environments, CAT, GSH-Px, and GR were dominant in the low background radiation culture, while SOD levels under low background radiation were expressed at lower levels than those under at standard background radiation, and GST activity showed no difference. Moreover, cell lines cultured in a low background radiation environment exhibited stronger scavenging capacity for organic and inorganic peroxides, weaker scavenging capacity for superoxide anions, higher hypoxanthine-guanine phosphoribosyl transferase (*hprt*) gene mutation frequency, and increased sensitivity to the mutagenic effect of γ-rays from _137_Cs sources (87). Replicating the experiment across a 10-month period, Fratini et al. (83) found equal SOD and CAT levels in cell lines from the two environments and significantly decreased GSH-Px levels (~4-fold lower than cells cultured in standard background). The activity of SOD, CAT, and GSH-Px did not change even if the cells affected by low background radiation were cultured in standard background radiation for 6 months. These authors also discovered significant differences in transcription levels (*GPx2* and *GPx4* gene) in cells from the two environments (83). In bacterial cells, *S. oneidensis* cultured over 50 h at the Waste Isolation Pilot Plant (underground laboratory) showed that oxidative stress-related genes, including *katB, recA*, and *SOA0154*, were upregulated by exposure to low radiation environments. The same research also found that *D. radiodurans* grown under low background radiation upregulated the expression of the *dnaK* gene, which is responsible for producing the heat shock protein HSP70. The upregulated genes of the two bacteria were restored after the recovery of radiation (82). A validation study found more upregulation of gene expression in *S. oneidensis* after exposure to low background radiation, including oxidative stress genes (*katB* and *oxyR*), DNA repair genes (*recA* and *lexA*), and other genes (*dnaK* and *SOA0154*). A replication study in *D. radiodurans* grown under low background radiation showed that the expression of the *lexA* and *dnaK* genes was upregulated, while the expression of genes related to oxidative stress (*dps* and *gapdH*) was decreased, and gene expression was restored after *D. radiodurans* was transferred from the low background to the standard background radiation (76).

## Future Research on the Underground Space Environment and Oxidative Stress

The above studies indicated that environmental factors in underground space, including hypoxia, toxic atmospheric particles, and low background radiation, may cause or upregulate oxidative stress in living organisms, as manifested by changes in oxidant/antioxidant indicators, gene oxidative damage and variation, as well as disease status. However, the findings of these studies are not entirely consistent, and the evidence is sometimes contradictory. Therefore, these findings need to be interpreted with caution, and further exploration is needed to elucidate the effect of these factors on the organism. Considering that most of the evidence suggests that these environmental factors have positive effects on organisms, we can take the following measures: (a) improve the ventilation facilities of the underground space environment to increase the oxygen supply and optimize air quality; (b) use low or non-toxic materials to reduce the release of toxic particles; (c) introduce an atmospheric filtration device or particle adsorption device; (d) promote the use of clean and renewable energy and reduce the exploitation of fossil fuels; (e) reduce the time spent by individuals in the underground space and encourage the use of dustproof masks; (f) avoid going into the deep space environment as much as possible; (g) elucidate the ultimate impact of low background radiation on organisms and introduce natural background radiation simulation devices; and (h) provide enough antioxidant supplements, such as vitamins C and E.

However, environmental factors of underground space include not only hypoxia, toxic atmospheric particles, and background radiation but also abnormal temperature, humidity, and atmospheric pressure, as well as special microorganisms, mental and psychological stress caused by confined space, and other unknown factors (2). In addition, the pathophysiological mechanism of underground space environmental factors in organisms is not limited to oxidative stress. Currently, studies have only focused on the correlation between hypoxia, toxic atmospheric particles and low background radiation, and oxidative stress in organisms, and no study has provided the correlation between other environmental factors in underground space and oxidative stress in organisms. Studies have shown that abnormal temperature and humidity, atmospheric pressure, radioactive elements, special microorganisms, and mental and psychological stress on the surface may cause oxidative stress damage to organisms (89–91). Therefore, we suggest that future studies should further identify and characterize the factors in the underground space environment that affect humans and living organisms, perform qualitative and quantitative analyses of the influence of these factors on humans and other organisms from the perspective of gene/protein/biological behavior and the level of cell/model organisms/human research, make full use of favorable factors, optimize the measures to prevent and treat exposure to unfavorable factors, and provide better protection for individuals entering the underground space. It is worth mentioning that our laboratory, experiments, and discipline (Deep-underground Medicine) are all in making rapid progress (2), but it seems to be insufficient, and we anticipate more resources will be involved in this issue.

## Conclusion

The effective exploitation and utilization of underground space and its resources is a worldwide trend, but it also involves the threats of the underground space environment to human health. Therefore, it is urgent to understand the effects of the underground space environment on human beings or other organisms. Current studies have only focused on the effects of a limited number of underground space environmental factors on oxidative stress and biological functions, and future research must be extended to other factors in the underground space environment and the underlying mechanisms and explore effective prevention and treatment strategies. This research will provide a solid foundation for humans to enter underground space.

## Author Contributions

HY and YG gathered and prepared all data. HY performed the writing of the paper and made the tables and figures. RZ performed the conceptualization and review of the paper. All authors contributed to the article and approved the submitted version.

## Conflict of Interest

The authors declare that the research was conducted in the absence of any commercial or financial relationships that could be construed as a potential conflict of interest.
