# Supplementary material for: Oxidative Stress From Exposure to the Underground Space Environment
Source: Front Public Health. 2020 Oct 21;8:579634. doi: 10.3389/fpubh.2020.579634 (PMC7609794; doi:10.3389/fpubh.2020.579634)
Supplement: Supplementary file 1 [file Table_1.DOCX]

Supplementary Material

**Supplementary Table 1** Subway systems and oxidative stress

| Author/ year | Location | Exposure matter | Exposure subjects | Oxidative indicators | Exposure effects |
| --- | --- | --- | --- | --- | --- |
| Karlsson et al., 2005 | Stockholm, Sweden | PM10 | Human lung epithelial cells (A549) | Intracellular  8-oxodG and DNA breakage | Intracellular  8-oxodG increased and oxidative DNA damage. |
| Bachoual et al., 2007 | Paris, France | PM10 | Murine macrophages (RAW 264.7) and C57BL/6 mice | Tissue homogenate and intracellular HO-1 | Tissue homogenate and intracellular HO-1 increased. |
| Lindbom et al., 2007 | Stockholm, Sweden | PM10 | Murine macrophages (RAW 264.7) | Intracellular  lipid peroxidation and ROS | Intracellular  lipid peroxidation and ROS increased. |
| Karlsson et al., 2008 | Stockholm, Sweden | PM10 | Human lung epithelial cells (A549) | Intracellular  ROS and oxidative DNA damage | Intracellular  ROS and oxidative DNA damage increase. |
| Grass et al., 2010 | New York, USA | PM2.5 | Subway workers | Isoprostane, protein carbonyls, 8-oxodG in blood and urine samples | No dose–response relationship between PM2.5 and the markers. |
| Kam et al., 2011b | Los Angeles, USA | PM (Coarse PM: 2.5−10μm; Fine PM: 2.5μm) | Alveolar macrophage | Intracellular  ROS | Intracellular  ROS increased significantly. |
| Jung et al., 2012 | Seoul, Korea | PM10 (Organic extract) | Human normal bronchial cells (BEAS-2B) and Chinese hamster ovary cells (CHO-K1) | Intracellular  ROS, SOD, CAT, oxidative DNA and chromosomal damage | Intracellular  ROS, SOD, CAT, oxidative DNA and chromosomal damage increased. |
| Kain et al., 2012 | Stockholm，Sweden | Not stated in detail | Human alveolar type II-like epithelial (A549) | Intracellular  ROS, DNA breaks, and 8-oxodG | Intracellular  ROS, DNA breaks, and 8-oxodG increased. |
| Loxham et al., 2013 | A subway station near a major European airport | PM (Coarse PM: 2.5-10μm; Fine PM: 2.5μm;  Ultrafine PM: 0.18μm) | Primary bronchial  epithelial cells (PBEC) | Intracellular  ROS | Intracellular  ROS increased. |
| Mehrdad et al., 2015 | Tehran, Iran | PM | Subway workers | Urinary 8-OHdG | Urinary 8-OHdG and oxidative DNA damage increased. |
| Spagnolo et al., 2015 | Italian | PM10 and PM2.5 | Non-small cell lung carcinoma cell (NCI-H727 cells) | Intracellular  ROS | Intracellular  ROS increased. |
| Loxham et al., 2015 | Mainline  underground  station,  Europe | PM (Coarse PM: 2.5-10μm; Fine PM: 2.5μm;  Ultrafine PM: 0.18μm) | Primary bronchial epithelial cells (PBECs) | Intracellular  ROS and HO-1 | Intracellular  ROS and HO-1 increased. |
| Gali et al., 2017 | Hong Kong, China | PM (Fine PM: < 2.5μm; Coarse PM: 2.5 -10μm) | Murine monocytic-macrophage cell (RAW 264.7) | Intracellular  ROS | Intracellular  ROS decreased relatively. |

PM, particular matter. 8-oxodG, 8-oxo-2'-deoxyguanosine. HO-1, heme oxygenase-1. ROS, reactive oxygen species. SOD, superoxide dismutase. CAT, catalase. GSH-Px, glutathione peroxidase. 8-OHdG, 8-hydroxy-deoxyguanosine.

**Supplementary Table 2** Underground mining and oxidative stress

| Author/ year | Location | Exposure subjects | Oxidative indicators | Study results |
| --- | --- | --- | --- | --- |
| Engelen et al., 1990 | Belgian | Coal workers | SOD, CAT, GSH-Px | The erythrocyte GSH-Px level of underground coal workers with pneumoconiosis imaging grading of 0/1 ~ 2/1 was significantly lower than that of underground coal workers without pneumoconiosis, and the antioxidant enzyme was significantly correlated with pneumoconiosis. |
| Schins et al., 1995 | Not stated | Coal workers | 8-oxodG, 8-oxodG/dG | The 8-oxodG/dG in lymphocytes did not differ between workers with CWP and workers without CWP, oxidative DNA damage in all workers was higher than in the non-exposed subjects. |
| Perrin-Nadif et al., 1996 | Lorraine, France | Coal workers | SOD, GSH-Px,  CAT, and total plasma antioxidant content | SOD activity in red blood cells of underground coal mine workers was significantly higher than that of the surface operation group, and there was no significant difference in the activity of CAT, GSH-Px and total plasma antioxidant content between the two groups. |
| Perrin-Nadif et al., 1998 | French | Coal workers | SOD, CAT, GSH-Px | There was no significant difference in the antioxidant enzyme activity between underground coal workers (without CWP) and surface coal workers, SOD activity was slightly decreased in the group of active underground workers with simple pneumoconiosis as compared with workers without CWP. |
| Nadif et al., 1998 | Lorraine，France | Coal workers | GSH-Px，CAT | There were significant correlations between occupational exposure to coal dust and erythrocyte GSH-Px activity and catalase activity. |
| Vallyathan et al., 2000 | Not stated | Coal workers | SOD, CAT, GSH-Px, lipid peroxidation | Antioxidant upregulation and lipid peroxides induction in bronchoalveolar lavage fluid in underground coal miners. |
| Zhai et al., 2002 | Guangxi Province, China | Coal workers | Genetic polymorphism of *MnSOD*, *GSTM1*, *GSTT1*, and *OGG1* | Cumulative coal dust exposure, rather than genetic polymorphism, was significantly correlated with CWP. |
| Nadif et al., 2003 | Lorraine，France | Coal workers | Genetic polymorphism of *LTA* and *TNF* | Genetic polymorphism and underground coal dust exposure were important components in the pathogenesis of CWP. |
| Altin et al., 2004 | Zonguldak, Turkey | Coal workers | MDA, SOD, GSH-Px | The serum MDA level, SOD and GSH-Px activity of underground workers were significantly higher than those of healthy subjects. The exposure time of underground worker was correlated with SOD, GSH-Px activity and MDA level. |
| Yucesoy et al., 2005 | Not stated | Ex-underground coal workers | Gene polymorphisms for *GSTP1*, *GSTT1* and  *MnSOD* | None of the individual *GST* or *MnSOD*  genotypes had a statistically  significant association with CWP. |
| Armutcu et al., 2007 | Zonguldak, Turkey | Wistar-Albino rats | MDA, SOD, GSH-Px, CAT, MPO, XO | Exposure to coal dust resulted in significantly increased oxidant parameters (MDA, XO activity) and MPO activity, and decreased antioxidant enzyme activity. |
| Ávila Júnior et al., 2009 | Santa  Catarina state, Brazil | Coal workers | TBARS, GSH, GSSG,  TG,  GST, GR, GSH-Px, CAT,  SOD | Compared with healthy subjects and other coal dust exposed subjects, the GSH-Px of underground coal workers decreased. |
| Wilhelm Filho et al., 2010 | Not stated | Coal workers | TBARS, PC, PT, AT, GSH, GST, GR, GSH-Px, CAT, SOD | After exposure to coal dust, TBARS and PC were increased, while PC, PT, AT, CAT, SOD, GSH-Px and GSH were decreased in underground miner. After supplementing with antioxidants, these indicators returned to normal. |
| Nardi et al., 2018 | Brazil | Quartz miners | MDA, vitamin C | The levels of MDA in plasma were significantly increased in coal miners. |

SOD, superoxide dismutase. CAT, catalase. GSH-Px, glutathione peroxidase. 8-OHdG, 8-hydroxy-deoxyguanosine. 8-oxodG, 8-oxo-2'-deoxyguanosine. GST, glutathione-S-transferase. MDA, Malondialdehyde. MPO, myeloperoxidase. XO, xanthine oxidase. PC, protein carbon. PT, protein thiols. AT, α-tocopherol. GR, glutathione reductase.
